# Defining and achieving permanency among older youth in foster care

**DOI:** 10.1016/j.childyouth.2018.02.006

**Published:** 2018-04

**Authors:** Amy M. Salazar, Kevin R. Jones, Jamie Amemiya, Adrian Cherry, Eric C. Brown, Richard F. Catalano, Kathryn C. Monahan

**Affiliations:** a Department of Human Development, Washington State University, 14204 NE Salmon Creek Ave., Vancouver, WA 98686-9600, USA; b Dorothy Day Social Work Program, University of Portland, 5000 N. Willamette Blvd., Portland, Oregon 97203-5798, USA; c Department of Psychology, University of Pittsburgh, 210 South Bouquet Street, Pittsburgh, PA 15260, USA; d Department of Public Health Sciences, University of Miami, 1120 NW 14th St., Suite 1014, Miami, FL 33136, USA; e Social Development Research Group, School of Social Work, University of Washington, 9725 3rd Ave. NE, Suite 401, Seattle, WA 98115, USA

**Keywords:** Foster care, Permanency, Child welfare, Aging out, Transition to adulthood

## Abstract

Permanency is a key child welfare system goal for the children they serve. This study addresses three key research questions: (1) How do older youth in foster care define their personal permanency goals? (2) How much progress have these youth made in achieving their personal permanency goals and other aspects of relational permanency, and how does this vary by gender, race, and age? and (3) What transition-related outcomes are associated with relational permanency achievement? Surveys were conducted with 97 youth between the ages of 14 and 20 currently in care. Over three-fourths of participants had an informal/relational permanency goal; however, only 6.7% had achieved their goal. Of eight additional conceptualizations of relational permanency assessed, the one associated with achievement of the highest number of key transition outcomes was *Sense of Family Belonging*. The transition outcomes with the most associations with permanency achievement were physical health and mental health. Relational permanency is a highly personal part of the transition process for youth in care, warranting personalized supports to ensure individual youths' goals are being addressed in transition planning. Permanency achievement may also provide a foundation for supporting youth in achieving other key transition outcomes.

## Highlights

• This paper explores relational permanency achievement among youth in foster care.
• Most youth reported having a permanency goal, but only 6.7% had achieved it.
• Achievement of some conceptualizations of permanency differed by race and gender.
• Physical and mental health were associated with permanency achievement.

## 1 Introduction

### 1.1 Background

Permanency is an important concept for children and youth with child welfare system involvement. The Adoption and Safe Families Act of 1997 laid the foundation for establishing permanency as one of three critical child welfare system goals, as it, along with child safety (Goal 2), is considered crucial for achieving child well-being (Goal 3). Permanency involves ensuring that children and youth having meaningful, enduring connections to a family or other long-term caring adults. In many cases permanency is a legal status, such as in the cases of adoption, legal guardianship, or reunification with one's biological family (i.e., *legal permanency*, or *formal permanency*). In fiscal year 2015, of the 243,060 children and youth who exited the foster care system, approximately half were reunified with their biological parents or primary caretakers, while an additional third were adopted, placed under legal guardianships, or were placed into the care of other relatives (US Department of Health and Human Services, 2016). Approximately 9% of youth emancipated from the foster care system (or “aged out”) without achievement of legal permanency. Youth who do not achieve legal permanency prior to aging out of the foster care system sometimes develop more or less structured *relational permanency* (also known as *informal permanency*) arrangements with relatives or other caring adults, such as those living with or receiving support from friends or family members but without a court order or other legally binding commitment. Relational permanency can come in many forms (for example, FosterClub's Permanency Pact tool offers 45 different types of supports caring adults may consider committing to providing youth, from providing a home for the holidays to being someone to talk to and discuss problems with; FosterClub, 2006), and can involve a wide variety of people, including biological relatives, non-kin supportive adults, people who have in the past been or continue to be paid service providers, peers, and romantic partners (Samuels, 2008).

Regardless of the type (legal or relational), permanency is a way to provide a secure foundation from which youth can engage meaningfully with the world around them. For older youth, it can provide the groundwork for preparing for and participating in adulthood and pursuing life goals. Studies have found that having meaningful, enduring relationships with caring adults is associated with a variety of positive adult outcomes for youth with foster care experience including increased postsecondary educational attainment (Salazar, 2012), having a bank account (Greeson, Usher, & Grinstein-Weiss, 2010), reduced risk of homelessness (Dworsky & Courtney, 2009), improved psychological well-being (Ahrens, DuBois, Richardson, Fan, & Lozano, 2008; Johnson, Pryce, & Martinovich, 2011), and improved physical health outcomes (Ahrens et al., 2008). Furthermore, having at least one stable relationship with a committed, caring adult has been found to be the single most common factor in youth who develop resilience (Harvard University's Center on the Developing Child, 2016). Taken cumulatively, permanency achievement may play a key role in youths' successful transitions to adulthood.

Older youth in care are much less likely than younger children to achieve legal permanency (Bass, Shields, & Behrman, 2004). Because of this, much of the focus in the case management for older youth in care often shifts from working toward reunification and/or adoption to preparing youth to live independently following their transition from foster care to adulthood. The Fostering Connections to Success and Increasing Adoptions Act of 2008 requires the development of personalized, youth-directed transition plans for youth in care 90 days prior to emancipation (and some programs support youth in developing and working on these plans much earlier than this). Transition plans often address several key transition areas, including employment, education, housing, physical and mental health, financial stability, and enduring connections to caring adults (i.e., relational permanency) if legal permanency is unlikely. Thus, youth often must think about what permanency means to them and what permanency-related goals they want to set for themselves, and more actively seek out this support from caring adults who may or may not be related to them. One study of youth who left care without legal permanence (Samuels, 2009) found that creating one's own personal definition of permanence was one strategy that youth used for dealing with the instability and “ambiguous loss of home” that they commonly experience during their journey through and exit from foster care. However, while there is strong support in the field for helping youth build relational permanency (at least in principle), little is known about what permanency goals older youth in care have for themselves, or how they perceive their progress toward permanency achievement. In addition, while the importance of caring adults in youths' lives for meeting many transition-related goals is well established, little is known about what types of permanency achievement are related to the achievement of other key transition-related outcomes.

In addition to age (older youth less likely to be adopted, more likely to achieve reunification or guardianship), race (White youth less likely to reunify, Black youth less likely to be adopted), disability status (youth with disabilities less likely to reunify or have guardianship, more likely to be adopted), kinship placements (those placed with relatives more likely to have guardianship, less likely to be adopted), placement with siblings (those placed with siblings more likely to achieve all types of legal permanency), establishing early stability while in care (associated with higher likelihood of reunification and adoption), runaway episodes (youth with episodes less likely to achieve any legal permanency type), and mental health challenges (youth with challenges less likely to be reunified or adopted) were all factors significantly associated with legal permanency achievement (Akin, 2011). These findings are consistent with earlier studies that have identified race, gender, age, and disability as factors affecting the duration of children's stay in the child welfare system (Becker, Jordan, & Larsen, 2007; Kemp & Bodonyi, 2002). However, it is less clear how these factors impact relational permanency achievement.

Finally, as was stated previously, permanency is one of many transition-related outcomes that are given attention to as part of the preparation of youth for the transition to adulthood. In fact, over the last several years, the Children's Bureau has begun collecting longitudinal data on cohorts of youth over the course of their transition from foster care to adulthood to assess how well youth are faring in these outcome areas through the National Youth in Transition Database. All of these outcomes (housing stability, educational attainment, employment, connectedness to caring adults, physical and mental health quality) are considered indicators of a successful transition to adulthood (Foster Care Independence Act, 1999). However, it is still unclear how youth-defined and other types of relational permanency achievement relate to various transition-related outcomes, or if achievement of certain conceptualizations of permanency, especially those that are relational rather than legal, are more strongly associated with positive transition outcomes than others.

### 1.2 Current study

This study addresses three key research questions: (1) How do youth transitioning from foster care to adulthood define their personal permanency goals? (2) How much progress do youth feel they have made in achieving their personal permanency goals and other conceptualizations of relational permanency during this transition, and how does this vary by gender, race, and age? (3) What transition-related outcomes are associated with various conceptualizations of relational permanency achievement among these youth? For Questions One and Two, we had no specific hypotheses as these questions were highly exploratory. For Question Three, we hypothesized that the farther along youth are in their permanency achievement, the more positively they are faring on other transition-related outcomes.

## 2 Method

### 2.1 Participants

Youth eligibility criteria for the larger study included (a) being between the ages of 14 and 22, and (b) being in the foster care system for at least six months in one urban county in the northeastern United States. This included youth who had already left the foster care system, but had spent at least six months in care in that county. Youth who met these criteria were identified by the county child welfare system. In order to facilitate a rapid data collection period, potential participants were contacted on the basis of proximity to the university where the majority of participant surveys occurred. There were two exceptions to this distance rule. First, group homes with eligible youth were invited as a whole to participate. Second, all individuals who had aged out of the system were eligible for enrollment regardless of distance from the research site.

A total of 330 youth (307 youth in care, 23 youth out of care) were contacted. Most (62.4%) of these individuals did not have valid contact information. Of those who were able to be reached (*n* = 124), 84% (*n* = 104) agreed to participate in the study. Basic demographic comparisons of the youth who refused to enroll in the study and those who agreed to participate revealed that the youth who refused to participate in the study were more likely to be in kinship care, but did not differ on age, gender, or whether they were currently in foster care.

This research was reviewed and approved by the Institutional Review Board of a large university in the northeastern United States. Because the county child welfare system is the legal guardian of these youth and gave permission for their participation, the Institutional Review Board provided a waiver of parent consent for this study, meaning that only youth had to agree to participate in order to be included in our sample. Surveys were conducted one-on-one by trained research staff and took approximately 1.5 h to complete. Youth received $100 for their participation.

In-person surveys were conducted with 104 youth between the ages of 14 and 22. For the current paper, only those currently in care (*n* = 97) were included in analyses; thus the age range for youth included in the current paper is 14–20. Table 1 shows demographics for these 97 participants. Race and ethnicity were restructured to three categories based on the largest groupings to simplify the analysis procedure: (a) Black non-Hispanic (53.6% of sample), (b) White non-Hispanic (20.6% of sample), and (c) other (which included everyone not falling into the first two categories; 21.6% of sample). Participants not reporting race and ethnicity (4.1% of sample) were left out of analyses involving race.

**Table 1:** Participant demographics.

Gender	Female:	50.5%
	Male:	49.5%
Race and ethnicity	Black Non-Hispanic:	53.6%
	White Non-Hispanic:	20.6%
	Other:	21.6%
	None reported:	4.1%
Age	14	7.2%
	15	12.4%
	16	19.6%
	17	20.6%
	18	16.5%
	19	16.5%
	20	7.2%
Mean age:	17.1	

### 2.2 Measures

#### 2.2.1 Permanency

Participants were first asked (yes, no, or don't know) whether they had a permanency goal, or a *personal goal for having a lifelong family*-*like connection*. If so, they were then given the opportunity in an open-ended question to describe their permanency goal. Next, they were asked to rate their current status in relation to achieving this permanency goal (1 = *nowhere near achieving this permanency goal* to 4 = *I have achieved this permanency goal*). Finally, participants were asked to rate their current status in relation to eight additional conceptualizations of permanency that emerged from an unrelated study's focus group with youth transitioning from foster care (Jim Casey Youth Opportunities Initiative, 2010): physical safety; psychological safety; unconditional love; concept that family will always be there; sense of family belonging; having people who believe in you; having grandparents for your children; and financial support. The response options were the same as those available for rating the status of their self-defined permanency goal.

#### 2.2.2 Employment experience

For employment experience, youth were asked (*yes* or *no*) whether they ever had a paying job.

#### 2.2.3 Arrests

For arrests, youth were asked (*yes* or *no*) whether they had been arrested in the past year.

#### 2.2.4 School commitment

Because the wide age range did not lend itself to a single appropriate educational completion milestone to test, school commitment was used to assess education status. School commitment was assessed using a 7-item scale adapted from the Communities That Care Youth Survey (Arthur, Hawkins, Pollard, Catalano, & Baglioni, 2002) that includes items such as, *Thinking back over the past year in school*, *how often did you try to do your best work*? In the Communities That Care studies, this scale was found to have α = 0.75 to 0.77 for junior high school students (Arthur et al., 2002).

#### 2.2.5 Mental health

As an indicator of mental health quality, depressive symptomatology was assessed using the PHQ-9 (the depression scale of the Patient Health Questionnaire), which has been found to have high internal reliability ranging from α = 0.86 to.89 in two large studies (Kroenke, Spitzer, & Williams, 2001). Each item is rated on a scale from 0 (*Not at all*) to 3 (*Nearly every day*), resulting in a possible overall score of 0 to 27. Established score cutpoints for depression severity are 5 = mild, 10 = moderate, 15 = moderately severe, and 20 = severe depression.

#### 2.2.6 Physical health

Physical health quality was assessed by asking youth *In general*, *how is your health*?, with ratings from 1 = *Poor* to 4 = *Excellent*. This item is from the Health-Related Quality of Life Scale's Healthy Days Core Module (CDC HRQOL-4; Centers for Disease Control and Prevention, 2015).

#### 2.2.7 Independent living preparation

Finally, as an indicator of preparation for independent living, youth were asked to rate how prepared they were to meet 5 types of goals (education, employment, personal health, financial, and housing), and how prepared they are to live on their own. These items are from the Midwest Evaluation of the Adult Functioning of Former Foster Youth (Courtney et al., 2007), and were combined by taking a mean of the responses from these 6 items, which ranged from 0 = *Not at all prepared* to 3 = *Very prepared*.

### 2.3 Data analysis procedure

Descriptive statistics were used to report whether youth had a permanency goal, and how far along they were in achieving that goal. Chi square tests were used to determine whether there were differences in having a permanency goal by gender, age, and race, while ANOVAs (for the 3 race category variable) and *t*-tests (for gender and age) were used to determine whether there were differences in permanency goal achievement by gender, age, and race. Due to the brief nature of participants' open-ended descriptions of their permanency goals, a very simple content analysis was conducted, in which responses were categorized by one researcher and reviewed by another researcher for agreement. Any disagreements were discussed and resolved, resulting in consensus-based categorizing. Finally, to test whether youths' degree of permanency achievement was related to the other transition outcome areas and in particular which conceptualizations of permanency were most closely related to these transition outcomes, 54 separate regression analyses (linear for those with continuous outcomes, binary logistic for those with dichotomous outcomes) were run with each type of permanency (self-defined and the 8 additional conceptualizations) as an independent variable and each transition outcome area as the dependent variable, each controlling for age, race, and gender.

## 3 Results

### 3.1 Personal permanency goals

Out of the 97 participants, 75 (77.3%) reported having a permanency goal. Table 2 shows permanency goal ownership by gender, age, and race. There were no significant differences among percentages of participants with permanency goals by these three factors.

**Table 2:** Permanency goals by gender, race, and age.

	All participants	Gender		Race and ethnicity			Age	
Have permanency goal?		Female	Male	Black Not Hispanic	White Not Hispanic	Other	Under 18	18 and older
Yes	77.3%	77.6%	77.1%	76.9%	70.0%	85.7%	70.7%	82.0%
No	14.4%	14.3%	14.6%	15.4%	15.0%	9.5%	15.5%	18.0%
Don't know/no response	8.2%	8.2%	8.3%	7.7%	15.0%	4.8%	13.8%	0.0%
(If has permanency goal = yes) How close to achieving this goal?								
Nowhere near achieving this goal	14.7%	13.2%	16.2%	10.0%	21.4%	16.7%	14.6%	14.7%
Have made some progress but still a long way to go	48.0%	42.1%	54.1%	55.0%	50.0%	27.8%	43.9%	52.9%
Have made a lot of progress but not quite there	30.7%	34.2%	27.0%	27.5%	28.6%	44.4%	29.3%	32.4%
Have achieved this goal	6.7%	10.5%	2.7%	7.5%	0.0%	11.1%	12.2%	0.0%

**Fig. 1:**
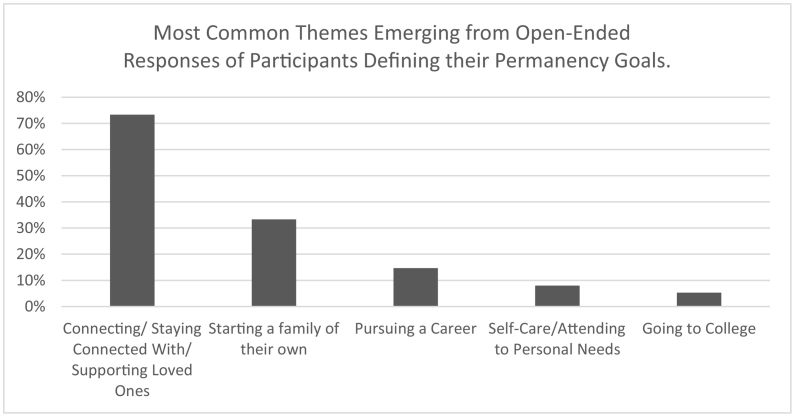
Most common themes emerging from open-ended responses of participants defining their permanency goals.

In an open-ended survey question, the 75 participants who identified as having permanency goals were asked to describe these goals to the interviewer. The 5 most common themes that emerged from this analysis, all of which were endorsed by at least 5% of the sample, can be found in Fig. 1. Each participant's response could have contained multiple themes. There were no significant differences in endorsement of these themes by participant age, race, or gender. The most common theme, connecting with and supporting loved ones, included responses referring to biological family members, foster families, and other loved ones not necessarily related to them, although most referred generically to “family” without specifying exactly who they were referring to. Some discussed specific ways of maintaining connections, such as through gatherings on holidays on birthdays. Others talked about supporting family members or moving back in with biological family members. For those endorsing the second most common theme, starting a family of their own, participants most commonly reported wanting to get married and/or have or adopt children. Those endorsing the career and college themes often discussed these permanency elements as part of a more detailed permanency plan that involved having a family and being able to support them. Finally, participants endorsing the self-care theme had permanency goals such as attending therapy, developing life skills, being able to have a healthy relationship, and more generally bettering oneself.

Those who reported having a permanency goal were also asked to rate how far along they were in relation to achieving this goal. Results can be seen in Table 2. Overall, only 5 (6.7%) of those who reported having a permanency goal said that they had achieved this goal. Three-fourths of youth (78.7%) had made some or a lot of progress, while 14.7% reported being nowhere near reaching their permanency goal. Table 2 shows goal achievement by gender, race, and age. There were no statistically significant differences in goal achievement by these characteristics.

### 3.2 Other permanency aspects

Youth were also asked to rate how far along they were in relation to achieving eight other conceptualizations of permanency that emerged from previous work with a separate sample of youth transitioning from foster care to adulthood (Jim Casey Youth Opportunities Initiative, 2010). Mean achievement scores (ranging from 1 = *Nowhere near achieving this* to 4 = *I have achieved this*) for each of the eight permanency conceptualizations were calculated. Table 3 shows the results; the first row includes participant ratings of achievement of their own personal goal, followed by the eight other permanency conceptualizations. The permanency types with the highest mean achievement score were *Having People who Believe in You* (*M* = 3.4, SD = 0.9) and *Physical Safety* (*M* = 3.4, SD = 0.8), while the permanency type with the lowest mean achievement score was participants' personal permanency goal (*M* = 2.3, SD = 0.8), followed by *Access to Financial Support* (*M* = 3.0, SD = 1.0).

**Table 3:** Participant ratings of their status with their personal permanency goal and eight additional permanency elements.

		Mean achievement status (SD) (not incl. don't know/no response)	Nowhere near achieving this (1)	Made some progress but still have a long way to go (2)	Made a lot of progress but not quite there (3)	I have achieved this (4)	Don't know/no response
Of those with a defined permanency goal	Participant-defined permanency goal	2.3 (0.8)	14.7%	48.0%	30.7%	6.7%	0.0%
Eight additional types of permanency, asked of all youth	Physical safety	3.4 (0.8)	2.1%	15.5%	22.7%	56.7%	3.1%
	Psychological safety/emotional security	3.2 (0.9)	5.2%	17.5%	24.7%	48.5%	4.1%
	Unconditional love, support, acceptance	3.3 (0.9)	6.2%	14.4%	25.8%	52.6%	1.0%
	Message that family will always be there no matter what	3.2 (1.1)	12.4%	10.3%	19.6%	53.6%	4.1%
	Sense of belonging with family, safe connections to past relationships	3.0 (1.1)	13.4%	15.5%	19.6%	44.3%	7.2%
	Have people who believe in you, hold your best interests at heart	3.4 (0.9)	4.1%	12.4%	21.6%	57.7%	4.1%
	Grandparents for your children	3.0 (1.2)	16.5%	11.3%	12.4%	45.4%	14.4%
	Help with or access to current and future financial support	3.0 (1.0)	9.3%	19.6%	21.6%	37.1%	12.4%

Differences in the achievement levels of these eight other conceptualizations of permanency by age, gender, and race were also assessed. Of the eight permanency types, only one, *Access to Financial Support*, differed by age range to a degree that reached statistical significance; those under age 18 reported a lower rate of achievement (*M* = 2.8, SD = 1.0) compared to youth aged 18 and older (*M* = 3.3, SD = 1.0; t(83) = −2.02, p = .046). Achievement of one permanency type, *Message that Family will Always Be There* (female *M* = 3.0, SD = 1.1; male *M* = 3.4, SD = 1.0), differed significantly by gender (t(91) = 2.22, p = .029), while achievement of *Psychological Safety* (female *M* = 3.0, SD = 1.0; male *M* = 3.4, SD = 0.9) approached statistical significance (t(91) = 1.83, p = .071). For both, males reported being farther along in their achievement of these types of permanency than females. Finally, one type of permanency, *Psychological Safety*, differed by race to a degree approaching statistical significance, with White participants (*M* = 3.7, SD = 0.7) reporting a higher rate of this type of permanency achievement than Black participants (*M* = 3.1, SD = 1.0) according to post hoc comparisons following a one-way ANOVA, *F* = (2, 87) = 2.81, p = .066).

### 3.3 Other transition-related factors associated with permanency achievement

Finally, we tested whether youths' degree of permanency achievement was related to several other key transition outcome areas. The results of these analyses can be found in Table 4. All associations were in the expected direction except for one: having employment experience was found to be negatively associated with *Psychological Safety*/*Emotional Support*.

**Table 4:** Relationships among types of informal permanency and transition-related outcomes, controlling for gender, race, and age.

	Descriptives		Permanency types																	
			Participant-defined permanence goal		Physical safety		Psychological safety/emotional security		Unconditional love, support, acceptance		Message that family will always be there		Sense of family belonging		Have people who believe in you		Grandparents for your children		Help with/access to financial support	
Dichotomous outcomes	Categories	%	Exp(B)	p	Exp(B)	p	Exp(B)	p	Exp(B)	p	Exp(B)	p	Exp(B)	p	Exp(B)	p	Exp(B)	p	Exp(B)	p
Employment: has employment experience	Yes	70.1%	1.16	0.704	0.87	0.705	**0**.**46**	**0**.**040**⁎	1.21	0.516	1.07	0.815	1.10	0.708	1.49	0.196	1.06	0.816	1.15	0.610
	No	29.9%																		
Arrested in the past year	Yes	12.4%	0.88	0.805	1.38	0.477	0.61	0.185	0.91	0.812	0.76	0.349	0.88	0.672	1.07	0.875	**0**.**47**	**0**.**020**⁎	0.60	0.131
	No	87.6%																		

Continuous outcomes	Ranges	M (SD)	Beta	p	Beta	p	Beta	p	Beta	p	Beta	p	Beta	p	Beta	p	Beta	p	Beta	p
Education: school commitment	1 = low to 5 = high commitment	3.5 (0.8)	0.19	0.129	−0.08	0.467	0.08	0.505	0.10	0.369	**0**.**21**	**0**.**066**^	0.09	0.448	**0**.**21**	**0**.**056**^	−0.05	0.696	0.12	0.306
Mental health: PHQ-9 depression score	0 = low to 27 = high	6.7 (5.3)	0.04	0.722	−0.07	0.500	−**0**.**26**	**0**.**012**⁎	−**0**.**30**	**0**.**003**⁎⁎	−**0**.**31**	**0**.**002**⁎⁎	−**0**.**26**	**0**.**013**⁎	−**0**.**19**	**0**.**064**^	−0.03	0.766	−0.11	0.334
Physical health rating	1 = poor to 4 = excellent	3.8 (1.1)	0.11	0.352	0.03	0.766	0.13	0.244	**0**.**21**	**0**.**045**⁎	**0**.**39**	**0**.**000**⁎⁎⁎	**0**.**24**	**0**.**023**⁎	**0**.**26**	**0**.**012**⁎	−0.03	0.786	**0**.**25**	**0**.**023**⁎
Preparation for independent living	0 = not at all to 3 = very prepared	2.1 (0.6)	**0**.**25**	**0**.**041**⁎	0.00	0.993	0.11	0.343	0.13	0.239	0.14	0.208	**0**.**29**	**0**.**008**⁎⁎	0.11	0.334	−0.02	0.896	0.12	0.297

Note 1: only includes youth aged 18 and older.

Numbers in bold indicate a relationship that was statistically significant at p < .1.

⁎⁎⁎ p = .000.

⁎⁎ p < .01.

⁎ p < .05.

^ p < .1.

Of the nine permanency types examined, the one significantly associated (at p < .05) with the highest number of key transition outcome areas (3 out of 6) was *Sense of Family Belonging*. All other permanency types were significantly associated with two or fewer transition outcome areas, and one, *Physical Safety*, was not associated with any transition-related outcomes. In terms of which transition outcome areas had the most associations with permanency types, physical health had statistically significant associations with 5 types of permanency, followed by mental health which had 4 significant associations. The remaining transition-related outcomes were significantly associated with two or fewer permanency types each.

## 4 Discussion

### 4.1 Summary of findings

This study explored how youth transitioning from foster care to adulthood think about their relational permanency goals, and how achievement of these goals is related to other transition-related outcomes. Youth rated their level of achievement of their own personal permanency goals as much lower than that of the other conceptualizations of permanency collected from focus groups with other youth in care. In addition, the degree of achievement of youths' personal permanency goal was found to be significantly associated with only one of the 6 transition-related outcomes assessed in the current study. Both of these findings may be due to the fact that several of the permanency goals laid out by youth were not particularly likely to be achieved for youth between the ages of 14 and 20 – goals such as having children or pursuing a career.

Similar to earlier findings from Samuels (2009), when providing their own definitions of permanency, participant responses in the current study were overwhelmingly reflective of relational permanency rather than legal permanency. Given that all youth in this study were still in foster care, it may be surprising that more youth did not express legal permanency goals (e.g., getting adopted, returning to one's biological family) more frequently or more explicitly. However, other than one participant whose goal was “to get home and stay home,” no other responses clearly and directly identified legal permanency as their key permanency goal.

While the cross-sectional nature of the data prevents causal inference, findings suggest that certain types of transition outcomes may be more sensitive to permanency achievement than others. For example, both physical and mental health were each associated with 4 to 5 of the different types of permanency. This suggests that transition-aged youth may experience psychological benefits from the experience of permanency in the context of loving, safe, and supportive relationships. Given the increase in mental health challenges experienced by young adults upon leaving foster care and the serious impact that impaired mental health can have on educational, employment, and social outcomes (Akister, Owens, & Goodyer, 2010), achieving relational permanency prior to aging out of foster care could serve as a preventive barrier for the development of some of these challenges.

It may be that some transition-related outcomes such as employment experience and preparation for independent living are not as important for some youth who have achieved permanency because that permanency in and of itself makes those outcomes less important to achieve; for example, if youth have a family who provides substantial, enduring care for them, the importance of being prepared to live independently may not be something that youth has to worry about. For most adolescents who have not been in foster care, the transition to adulthood often comes with substantial support from family. A 2016 Pew Research Center study (Fry, 2016) found the most common living arrangement for young adults aged 18 to 34 to be with their parents (32.1% of young adults). Relatedly, another study (Pew Research Center, 2015) found that 61% of adults in the United States had helped their adult children financially in the past year, 48% had helped their adult children with child care, and 39% had helped with errands, housework, or home repairs. For this large subset of the young adult population, living independently is not the reality or necessarily even a goal. It is possible that a similar pattern is happening for at least a small subset of youth in care who are able to secure similar types of supportive relationships with caring adults while they are still in care, making these transition goals less important than they are for other foster youth preparing to transition to adulthood.

### 4.2 Implications and next steps

While *fully achieved* was the most common response to each of the eight permanency types defined by youth in other studies, <10% of participants felt they had yet fully achieved their own personal permanency goal. Taken cumulatively, permanency-focused supports for youth transitioning from foster care would likely benefit from paying attention to youth-specific goals to ensure that individual youths' goals are being addressed in transition planning.

The relationship between permanency achievement and health outcomes is another area deserving more attention in research and practice. The safety and security of achieving permanency likely reduces the psychological stress and uncertainty that older youth in the foster care system experience, and effective treatment of mental health issues is also likely to make permanency goals more achievable. In practice, that means possibly working from both ends—supporting mental health to achieve permanency and supporting permanency to improve mental health. Better mental health directly contributes to better physical health (Kolappa, Henderson, & Kishore, 2013) and can mitigate the harm caused by mental health-related outcomes such as substance use (Deykin, Levy, & Wells, 1987), self-harm (Skegg, 2005), and risky sexual behaviors (Ramrakha, Caspi, Dickson, Moffitt, & Paul, 2000). Considered together, investment in the dual goals of achieving permanency and improving mental health outcomes for transition-aged youth in foster care should be a top priority. Further, since causal relationships between permanency and health were not able to be determined in this study, future studies using longitudinal and experimental designs would be significant contributions to the field.

Race and gender have been identified as significant predictors of risk tolerance, largely independent of wealth, macroeconomic conditions, and other contextual factors, with African Americans and females demonstrating lower tolerance for risk (and therefore lower levels of psychological safety) than males and people of other races (Sahm, 2014). Further, females and Black males experience less psychological safety when they encounter situations in which their identities are directly devalued, or when institutional norms are not aligned with their identities (Wanless, 2016). Disproportionality in out-of-home care and lower rates of timely permanency and adoption (Smith, McRoy, Freundlich, & Kroll, 2008), for example, may create the conditions for black youth to experience less psychological safety in the foster care system. Adolescent girls in foster care are three times more likely than boys to have been molested and five times more likely to have been raped (Salazar, Keller, Gowen, & Courtney, 2013), and this sexual trauma could further contribute to gender differences in reports of psychological safety.

For Black and female youth in foster care who experience less psychological safety than their White/male peers, it may be useful for professionals and researchers to explore the potential for trauma-informed practices to address the ongoing effects of abuse, racism, sexism, sexual victimization, and other life events associated with trauma symptoms and PTSD. Trauma-informed approaches have been successful in reducing mental health crises, violence, and self-harm among women who are incarcerated (Benedict, 2014)—a group with similarly elevated rates of trauma experience. Gender-responsive trauma interventions that “reflect the realities of women's lives,” and include, “program development, content, and material that reflects an understanding of the realities of women's and girls' lives and is responsive to their strengths and challenges,” have been effective in helping reduce substance use and trauma symptoms (Covington, Burke, Keaton, & Norcott, 2008, p. 390). Trauma-informed services for African-American youth are severely lacking, which means that, “African-American children and youth, who are among the most likely members of our society to be exposed to trauma, are also among the least likely to receive the services that could prevent the development of trauma-related emotional and behavioral difficulties” (National Child Traumatic Stress Network, 2016, p. 3). Given the extremely high rates of trauma exposure and PTSD among older youth in foster care generally (Salazar et al., 2013), trauma-informed interventions may offer particularly appropriate and responsive opportunities for health and healing among the most vulnerable youth in the foster care system.

An additional valuable next step would be to assess how permanency perceptions, conceptualizations, and achievement change after youth have left the child welfare system. It is possible that once youth leave the system their perceptions of their permanency situations may shift, especially if they are finding themselves with fewer supportive adults to reach out to. Assessing how permanency perceptions change before and after leaving care could help practitioners understand how to better prepare youth for that transition, especially if youths' permanency achievement perceptions change drastically following this transition.

### 4.3 Limitations

One notable limitation of this study is the cross-sectional nature of the data, which prevents the ability to make causal inferences regarding the impact that permanency achievement may be having on the achievement of transition-related outcomes. Second, the short study time frame leading to the necessity of giving preference to potential participants based on their proximity to the university limits generalizability. Third, this study includes youth impacted by one county child welfare system; youth from other systems may show different relationships between permanency and transition-related outcome achievements. Finally, the need to prioritize youth closer to the data collection site for participation may have resulted in skewed findings.

## 5 Conclusion

Youth transitioning from foster care to adulthood are in a unique position that often requires them to actively think about their permanency goals and proactively reach out to caring adults in their lives to reach these goals. Support efforts should incorporate youth-defined conceptualizations of permanency in order to have the best chance at meaningful goal achievement. In addition to its inherent importance, permanency achievement may also provide a foundation for supporting youth in achieving other key transition-related outcomes.

## Conflicts of interest

There are no conflicts of interest to report for this study.
